# Nanomedicine in Ophthalmology: From Bench to Bedside

**DOI:** 10.3390/jcm13247651

**Published:** 2024-12-16

**Authors:** Binapani Mahaling, Namrata Baruah, Aumreetam Dinabandhu

**Affiliations:** 1 Schepens Eye Research Institute, Harvard Medical School, Boston, MA 02114, USA; 2 Emory National Primate Research Center, Emory University, Atlanta, GA 30329, USA; nbaruah@emory.edu; 3 Wilmer Eye Institute, The Johns Hopkins University School of Medicine, Baltimore, MD 21231, USA; aumreetamd@gmail.com

**Keywords:** nanomedicine, ocular diseases, clinical trials, macular degeneration, diabetic retinopathy, dry eye disease, nanotechnology

## Abstract

Ocular diseases such as cataract, refractive error, age-related macular degeneration, glaucoma, and diabetic retinopathy significantly impact vision and quality of life worldwide. Despite advances in conventional treatments, challenges like limited bioavailability, poor patient compliance, and invasive administration methods hinder their effectiveness. Nanomedicine offers a promising solution by enhancing drug delivery to targeted ocular tissues, enabling sustained release, and improving therapeutic outcomes. This review explores the journey of nanomedicine from bench to bedside, focusing on key nanotechnology platforms, preclinical models, and case studies of successful clinical translation. It addresses critical challenges, including pharmacokinetics, regulatory hurdles, and manufacturing scalability, which must be overcome for successful market entry. Additionally, this review highlights safety considerations, current marketed and FDA-approved nanomedicine products, and emerging trends such as gene therapy and personalized approaches. By providing a comprehensive overview of the current landscape and future directions, this article aims to guide researchers, clinicians, and industry stakeholders in advancing the clinical application of nanomedicine in ophthalmology.

## 1. Introduction

Ocular diseases pose a significant public health challenge, affecting millions of individuals globally. In 2020, it was estimated that 43.3 million people worldwide were blind, while 295 million had moderate to severe vision impairment, and an additional 258 million experienced mild vision impairment [1]. Conditions such as cataract, glaucoma, refractive error, age-related macular degeneration (AMD), and diabetic retinopathy (DR) are leading causes of visual impairment and blindness [2]. For instance, cataract, AMD, and DR are responsible for vision loss in the elderly and diabetic populations, respectively, while glaucoma remains a major contributor to irreversible blindness [2,3]. Despite advances in pharmaceutical and surgical treatments, conventional therapies often face challenges such as poor drug penetration, rapid clearance, and low patient compliance [4,5]. The complex anatomy and physiology of the eye, characterized by barriers like the cornea, blood–retina barrier, and vitreous body, limit the efficacy of topical, systemic, and even some intravitreal therapies [4,6]. These limitations underscore the need for innovative drug delivery strategies that can enhance therapeutic outcomes and improve patient quality of life.

Nanomedicine has emerged as a promising approach to address the challenges associated with ocular drug delivery. By leveraging nanoparticles, liposomes, micelles, and other nanoscale carriers, researchers can improve drug stability, achieve controlled release, provide non-invasive modes of administration and enhanced patience compliance, and target specific ocular tissues [4,6,7]. Nanotechnology-based delivery systems have the potential to increase drug bioavailability, reduce dosing frequency, and minimize systemic side effects [4]. Additionally, they can enable the delivery of new therapeutic modalities like gene therapy and ribonucleic acid (RNA)-based treatments, which hold great promise for retinal diseases and other complex conditions [8,9,10]. This review aims to assess the progress made in translating nanomedicine from research to clinical practice for ocular diseases. It highlights the key technological platforms, reviews the current clinical status of nanomedicines/drugs and investigational products, and addresses the main challenges in clinical translation. By offering insights into both successful and failed attempts, this article seeks to guide future research efforts and promote collaboration among scientists, clinicians, and regulatory bodies in the field of ocular nanomedicine.

## 2. Overview of Nanomedicine in Ophthalmology

### 2.1. Key Nanotechnology Platforms

**Figure 1:**
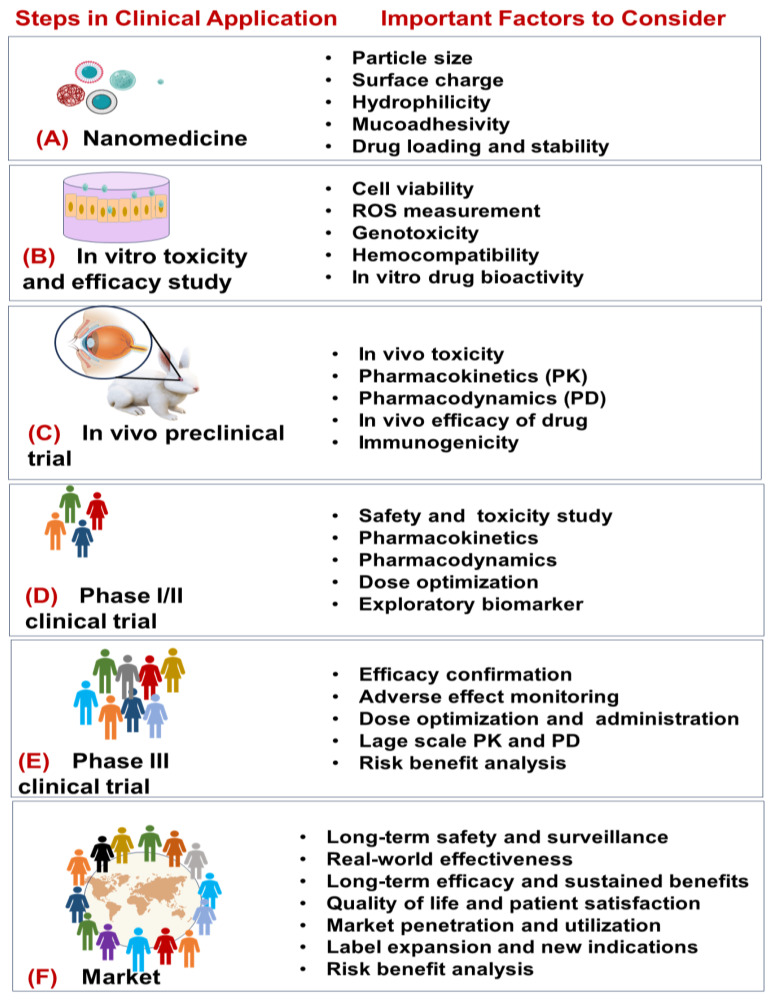
A schematic representation of the sequential steps in the clinical application of nanomedicines, highlighting important factors to consider. (**A**) Nanocarriers are fabricated and characterized by specific physicochemical properties during the initial development phase. (**B**) In vitro studies assess toxicity and efficacy by testing various factors for validation. (**C**) Preclinical testing is conducted in suitable in vivo model systems based on the targeted ocular disease. (**D**) Phase I/II clinical trials evaluate safety, toxicity, and preliminary efficacy and optimize dosing in a small population. (**E**) Phase III clinical trials confirm efficacy and dosing and identify any adverse effects in a larger population. (**F**) Finally, the nanomedicine is marketed for therapeutic use in real-world settings.

The field of nanomedicine has introduced a variety of nanocarriers such as nanoparticles, nanospheres, liposomes, micelles, niosomes, nanosponges, nanosuspensions, nanoemulsions, cubosomes, nanogels, nanomedicine-laden contact lenses, carbon nanotubes, nanoprobes, nanobelts, nanoantennae, and extracellular vesicles designed to enhance drug delivery for ocular diseases which we have reviewed in great detail [4,6]. These nanoscale platforms help to overcome the challenges of delivering therapeutics to the eye, such as ocular barriers, limited drug penetration, short retention time, and rapid clearance from the ocular surface [11,12,13,14,15,16]. Nanomedicines offer significant advantages over conventional therapies by protecting drugs from degradation, enhancing their solubility, and enabling sustained release, targeted drug delivery, and the release of drugs under various stimuli. These features collectively improve drug bioavailability and enhance therapeutic outcomes, especially for chronic diseases like DR, glaucoma, and AMD [7,17,18,19,20,21]. As a result, they represent a critical area of research and development in the journey toward the clinical translation of nanomedicine in ophthalmology (Figure 1).

### 2.2. Current Clinical Status

Several nanomedicine products have gained the U.S. Food And Drug Administration’s (FDA) approval for the treatment of various ocular diseases by addressing the limitations of traditional therapies through improved drug delivery mechanisms. These approved products are designed to enhance the stability, bioavailability, and therapeutic effectiveness of drugs while reducing side effects and improving patient compliance [22,23,24]. Some of the key FDA-approved nanomedicines for ocular use are included in Table 1.

Beyond the FDA-approved products, a range of innovative nanomedicine formulations are currently in advanced stages of development, aiming to improve the treatment landscape for various eye diseases. These systems often include nanoparticles, sustained-release implants, micelles, or gene therapy vectors designed to treat conditions like AMD, diabetic macular edema (DME), glaucoma, and more. Below are examples of recent clinical trials in various phases (Table 2) which focus on novel drug delivery systems that have advanced beyond preclinical studies.

## 3. Preclinical Models for Ocular Diseases

The development of nanomedicines for ocular diseases involves rigorous testing in preclinical models to ensure their safety, efficacy, and mechanism of action before advancing to clinical trials. These models help mimic the human eye’s complex anatomy and pathology, providing crucial insights into how nanomedicines interact with ocular tissues [47,48,49]. The different types of models explored for testing nanomedicines are in vitro cell culture, ex vivo organoid culture, 3D tissue culture, and in vivo animal models for ocular diseases.

### 3.1. In Vitro Model

**Figure 2:**
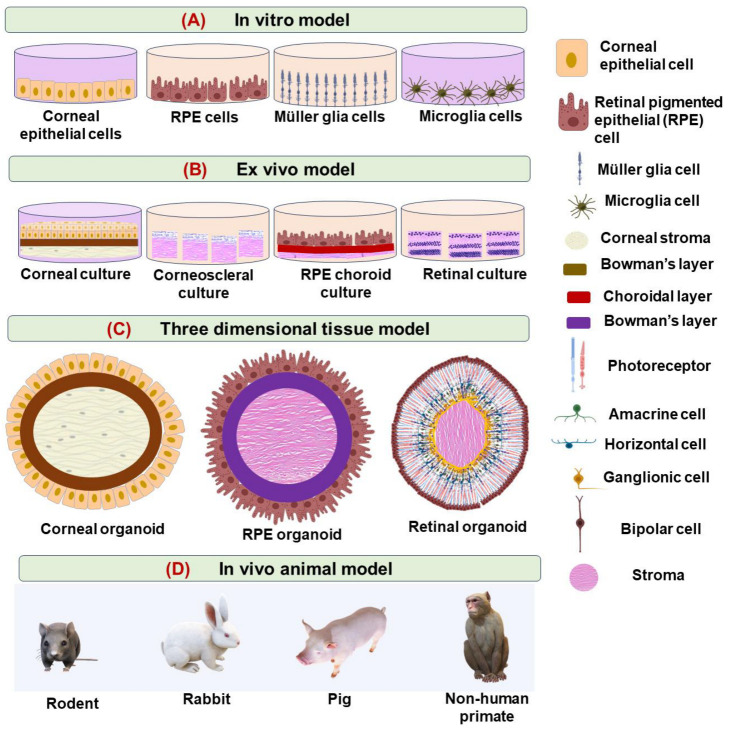
A schematic representation of preclinical models for ocular diseases. (**A**) In vitro two-dimensional (2D) cultures of ocular cells are used for initial screening of nanomedicines/drugs to assess their effectiveness, toxicity, and bioactivity. (**B**) Ex vivo cultures of ocular tissues are employed to test nanomedicine/drug formulations and optimize concentrations under controlled conditions. (**C**) Three-dimensional (3D) ocular tissue models, such as organoid cultures, are used for nanomedicine/drug development and validation prior to animal testing. (**D**) In vivo animal models are utilized to study the pharmacokinetics and pharmacodynamics for a more accurate assessment of nanomedicine/drug delivery systems.

An in vitro two-dimensional (2D) cell culture model (Figure 2A) is used in the initial steps to screen the effectiveness, toxicity, and bioactivity of the nanomedicines before progressing to animal models [4,7,11]. These models are critical for understanding the interaction of nanoparticles with ocular cells at a molecular level. Both primary and immortalized cells are explored to study the effectiveness, toxicity, and bioactivity of the nanomedicines. The different cells and cell lines explored are human corneal epithelial cells (HCEs), a rabbit corneal epithelial cell line (SIRC), rabbit corneal keratocytes, retinal pigment epithelial cells (ARPE-19), human conjunctival epithelial cells (WKD), Muller glia (MIO-M1), Microglia (BV-2), and macrophages (RAW-267) [4,50,51,52].

Corneal, conjunctival, and retinal epithelial cell cultures help study the penetration and toxicity of nanoparticles across the epithelial barrier. They are especially useful in evaluating formulations aimed at delivering drugs to the anterior or posterior segment of the eye. These cells are also used to determine the toxicity of the nanomedicines [48,53]. In vitro cell-based models are advantageous because they are simple, quick to develop, cost-effective, and highly reproducible. Additionally, they offer valuable insights into the underlying mechanisms of drug interactions and effects. However, they do not fully mimic the complex three-dimensional structure and microenvironment of the human cornea/conjunctiva/retinal pigmented epithelium [48].

In vitro glial cell cultures are valuable for studying the interaction of drugs/nanoparticles with the central nervous system and ocular environments. They provide insights into nanoparticle uptake, immune responses, and therapeutic effects. Müller cells, microglia, and astrocytes are the primary glial cell types used in these studies [54,55,56,57]. Müller cell cultures are used to explore how nanoparticles can be engineered for drug delivery to modulate oxidative stress and support neuroprotection in retinal diseases. For example, studies have investigated the use of solid lipid nanoparticles to deliver anti-oxidant drugs to Müller cells in vitro. These cultures are useful for evaluating nanoparticle uptake, cytotoxicity, and the release of inflammatory mediators [55]. Microglial cultures help assess the impact of nanoparticles on immune responses in the central nervous system. For example, studies using BV2 microglial cell lines have examined the uptake of FA–CS/PMSN/miR-223 nanoparticles and their potential for targeted delivery for retinopathy. These cultures are also used to study how nanoparticles influence microglial polarization cytokine secretion [56]. Astrocyte cultures are employed to evaluate the effects of nanoparticles on maintaining the integrity of the blood–retina barrier (BRB) and their influence on glial reactivity. Primary astrocytes and immortalized lines provide a platform for assessing nanoparticle-induced oxidative stress and neuroinflammation. For instance, research has explored the interactions between gold nanoparticles on astrocytes and endothelial cells to understand the risks of neurotoxicity [57].

### 3.2. Ex Vivo Model

An ex vivo model (Figure 2B) serves as an intermediate step between in vitro cell cultures and in vivo animal studies, offering a more realistic environment to study drug delivery in ocular tissues. These models use tissues extracted from animals or humans to preserve the natural architecture and physiological functions of the eye, enabling researchers to study drug penetration, distribution, and effects in a controlled setting [48,58,59]. This approach can be particularly valuable for testing nanomedicine formulations and drug delivery systems targeting specific regions of the eye [59,60]. Commonly used ex vivo models in ocular research are corneal, scleral, or retinal pigmented epithelial cell–choroid permeability models [60,61,62].

Corneal tissues, often from animals like bovine, rabbits, or pigs, are used to study drug permeability through the corneal epithelium. These models are particularly useful for evaluating topical formulations such as eye drops and nanoparticle-based gels [60,63,64,65,66]. Assessing the ability of nanoparticles to penetrate the corneal barrier and reach deeper ocular tissues can help optimize the formulation for better bioavailability [65]. An ex vivo study offers a more accurate prediction of corneal penetration compared to cell monolayers. It also allows for the testing of drug penetration through all layers of the cornea (epithelium, stroma, and endothelium) [66]. However, corneal tissues degrade over time, making long-term studies challenging. Additionally, there are differences between animal and human corneas that can affect the translation of results [66].

Scleral tissues, typically from bovine, pigs, or human donors, are used to study the permeability of drug formulations targeting the posterior segment of the eye, such as the retina and choroid [67,68,69]. These can also be useful to test formulations like nanoparticles, microparticles, or sustained-release implants intended for scleral delivery or subconjunctival administration. It provides insights into how these formulations diffuse through the sclera to reach deeper ocular tissues [67,70]. The scleral permeability test helps to evaluate the suitability of trans-scleral routes for drugs targeting the posterior segment, potentially reducing the need for invasive procedures like intravitreal injections [12,70]. However, ex vivo scleral models have some limitations as the variability in scleral thickness between species can influence the results, and the lack of active blood flow in ex vivo conditions may affect drug distribution dynamics.

Retinal explants from animals, such as rodents, pigs, rabbits, and bovine, are used to study the effects of drugs on the retina. These models preserve the layered structure of the retina and maintain the relationship between the retinal pigment epithelium (RPE) and photoreceptors [71,72,73,74,75]. It could be useful to test the penetration and efficacy of intravitreal injections or nanoparticles designed to target retinal cells. They are also used for evaluating drug-induced toxicity and neuroprotective effects of drugs and nanomedicines [71,75,76]. The retina explant culture provides insights into the interactions between nanoparticles and the complex layers of the retina, which are crucial for targeting conditions like AMD or DR [75,76]. However, the retinal explant model has some limitations, as it can only be kept viable for a limited time, making long-term studies challenging. Additionally, these models do not replicate systemic influences such as immune responses.

Ex vivo models offer several advantages compared to in vitro and in vivo systems: (i) Ex vivo models maintain the natural structural complexity of ocular tissues, offering a more accurate representation of drug–tissue interactions compared to in vitro models. This makes them particularly useful for studying drug penetration and distribution within the different layers of ocular tissues [63,71]. (ii) These models are less expensive than in vivo studies, as they do not require maintaining live animals. They can also reduce the need for animal use in early-stage testing, aligning with ethical considerations. (iii) By studying isolated tissues, researchers can control specific conditions, such as drug concentration and exposure time, to better understand the mechanisms of drug action without the confounding variables present in living organisms. (iv) Using human donor tissues when available can provide a closer approximation to human ocular physiology, improving the predictive value of these models for clinical translation.

### 3.3. Three-Dimensional Tissue Model

A three-dimensional (3D) ocular tissue model (Figure 2C) provides a more accurate representation of the organism’s environment compared to two-dimensional cultures. In these models, cells grow in all directions, closely mimicking tissue architecture in vitro and serving as a valuable complement to 2D cell cultures [48,77]. Advanced 3D models, such as 3D engineered ocular tissue, organoids, spheroids, and organ-on-a-chip mimic the multi-layered structure of the human eye better than simple cell cultures. These models can reproduce the interactions between different cell types, such as endothelial and epithelial cells, under controlled conditions [78,79].

#### 3.3.1. Organoid Model

The 3D organoid cornea is typically developed from stem cells or progenitor cells and is cultured to form layers similar to the native cornea, including the epithelium, stroma, and endothelium. The 3D organoid model (Figure 2C) allows for a more realistic study of corneal development, disease modeling, drug testing, and regenerative medicine applications compared to traditional 2D cell cultures. It closely replicates the natural microenvironment, making it an important tool for ophthalmic research [79,80].

A 3D retinal organoid is a lab-grown, three-dimensional structure that replicates key features of the retina. These organoids are usually derived from pluripotent stem cells, which are cultured under specific conditions to develop into various retinal cell types, including photoreceptors and ganglion cells. It offers a more physiologically relevant model for studying retinal development, degenerative diseases, and potential therapies, such as gene and cell therapies. Unlike 2D cultures, these organoids can better mimic the spatial organization and cell–cell interactions found in the human retina, making them a powerful tool for vision research and drug testing [80,81].

RPE organoids are generated from pluripotent stem cells that are directed to differentiate into RPE cells, forming a layered structure with polarized, pigmented epithelial cells on the surface and a collagen matrix-filled core. This structure closely resembles the native RPE in vivo, providing a more physiologically accurate model for studying RPE function and retinal diseases such as AMD. RPE organoids offer a more faithful representation of the complex structure and function of the RPE compared to traditional 2D cultures, making them valuable for ophthalmic research and drug development [80].

Organoid models can be valuable for evaluating drug penetration, release kinetics, and nanoparticle retention across different layers of the eye. However, they have certain limitations. While they offer a closer approximation to human eye physiology, they still lack the dynamic blood flow and immune responses found in living organisms.

#### 3.3.2. Spheroid Model

Spheroid models are three-dimensional clusters of cells that mimic the architecture and microenvironment of eye tissues, such as the retina, tear film, cornea, or RPE. These models are created by culturing cells in a way that allows them to self-assemble into spherical structures, providing a more physiologically relevant environment than traditional 2D cultures [78]. Spheroid models are useful for studying cell–cell interactions, tissue-specific responses, drug delivery, and toxicity in a more realistic context. They also serve as valuable tools for investigating the effects of new therapies on eye tissues, including drug penetration and sustained release. However, like other in vitro models, they have limitations, such as lacking the vascular and immune components present in living systems [78].

Three-dimensional spheroids composed of choroid–retinal vascular endothelial cells serve as a valuable in vitro model for studying diabetic retinopathy. These spheroids mimic the three-dimensional structure and microenvironment of the retinal vasculature more effectively than traditional 2D cultures, allowing for better representation of cell–cell and cell–matrix interactions. This model can be used to investigate the pathophysiological changes associated with diabetic retinopathy, such as abnormal blood vessel formation, permeability changes, and oxidative stress. Additionally, it provides a platform to test therapeutic strategies, including drug delivery and efficacy, in a controlled setting that closely mimics the in vivo environment of the eye. However, like other in vitro models, these spheroids do not fully replicate the complex interplay with the immune system and systemic factors present in living organisms [82].

Spheroid models of the ocular surface and tear film system, including the lacrimal gland, provide a three-dimensional in vitro platform to study the physiology and pathology of these critical components of the eye. These models are formed by culturing epithelial cells with endothelial cells and mesenchymal stem cells (MSCs) or conjunctival epithelium and lacrimal gland cells, allowing them to self-organize into spheroids that mimic the natural structure and function of ocular surface tissues. The spheroid model closely replicates cell–cell interactions, secretion dynamics, and structural organization of the tear film, making it ideal for studying tear production with both aqueous and mucin layers. It also allows for the investigation of conditions like dry eye syndrome, lacrimal gland dysfunction, and other disorders affecting tear production and stability [83,84]. Moreover, this model can be used to assess drug delivery, drug retention, and the effects of new therapies on tear film stability and ocular surface integrity. However, spheroid models, while providing a more realistic microenvironment than traditional 2D cultures, lack the complete anatomical complexity of the eye, including the vascular and immune components that play a crucial role in the eye’s response to injury and disease.

Cultivated cell spheroid transplantation is a widely studied approach for promoting tissue regeneration. Chitosan biomaterials have been shown to facilitate keratocyte aggregation and the formation of multicellular spheroids. These keratocyte spheroids are capable of maintaining their cellular phenotype, secreting collagen matrix, and improving graft retention, indicating their strong potential for repairing stromal tissue defects and reducing corneal haze or edema. This makes keratocyte spheroids a promising platform for testing nanomedicine-based therapies, such as targeted drug delivery systems or nanoparticle-mediated treatments. Their three-dimensional structure may play a crucial role in influencing nanoparticle retention and controlling drug release kinetics within the corneal microenvironment [85].

#### 3.3.3. Three-Dimensional Tissue Engineering Models

Three-dimensional tissue engineering models for the eye are advanced in vitro systems designed to replicate the complex structure and function of ocular tissues, including the cornea, retina, and other components. These models are developed using biocompatible scaffolds, hydrogels, or decellularized matrices seeded with specific cell types, such as corneal epithelial cells, retinal pigment epithelium (RPE) cells, or retinal neurons. The cells grow and organize into layered structures that closely mimic the natural architecture of the eye [77,86]. Three-dimensional tissue-engineered models allow for a more accurate study of cellular behavior, tissue interactions, and the effects of drugs on ocular tissues. They provide a controlled environment to investigate various eye conditions, such as AMD, diabetic retinopathy, corneal scarring, and glaucoma [86,87,88]. Additionally, they can be used to study drug delivery systems, gene therapy, and regenerative approaches.

These models offer significant advantages over traditional 2D cultures by more accurately reproducing the physiological conditions of the eye. They enable the study of cell–cell and cell–matrix interactions, as well as mechanical properties like tissue stiffness. However, they still lack the systemic interactions, blood flow, and immune response that are present in living organisms, which can limit their ability to fully predict therapeutic outcomes. Despite these limitations, 3D tissue-engineered models are crucial tools for advancing research in ophthalmology and developing new treatments for eye diseases.

### 3.4. In Vivo Model

An in vivo animal model (Figure 2D) provides a more holistic understanding of how a nanomedicine interacts with the eye’s anatomy, pharmacokinetics (absorption, distribution, metabolism, and excretion), and pharmacodynamics (biological effects). They are advantageous as they offer a more accurate assessment of nanomedicine but are subjected to ethical considerations and regulatory hurdles. These models are crucial for simulating the complexities of human ocular diseases. The different animal models explored for preclinical trials of ocular nanomedicines are mice, rats, rabbits, pigs, non-human primates, and occasionally zebra fish.

#### 3.4.1. Mouse Model

Mouse models are extensively used in ocular research to study various eye diseases and test potential therapies due to having several advantages: they are amenable to genetic manipulation, have a range of available knockouts and knock-ins, have a shorter lifespan, are easy to handle, are cost-effective, and mimic key aspects of human disease conditions. Mouse models are particularly suitable for investigating the pathogenic mechanisms of inherited human diseases. Common mouse models for ocular diseases, including genetic disorders, are used to assess drug release profiles, toxicity, efficacy, and retinal targeting of nanomedicines through systemic or local delivery methods such as intravitreal injections. Streptozotocin (STZ)-induced diabetic mice models develop hyperglycemia and exhibit pathological changes similar to those seen in human diabetic retinopathy, including retinal vascular alterations and inflammation [89]. Ccl2 and Cx3Cr1 deficiency mice models display features resembling dry AMD, such as retinal pigment epithelium (RPE) dysfunction, drusen-like deposits, and progressive photoreceptor degeneration [90]. The DBA/2J mice strain develops elevated intraocular pressure (IOP) and optic nerve degeneration, making it a valuable model for studying glaucoma mechanisms and potential therapies [91]. Experimental autoimmune uveitis (EAU) induced in C57BL/6 mice is immunized with retinal antigens to induce uveitis, allowing researchers to explore its pathophysiology and evaluate potential treatments [92]. Rho−/− (RHO mutant) and Pde6b−/− mice models exhibit progressive photoreceptor degeneration and can be utilized to test gene therapies and retinal implants [93,94]. Mouse models with induced corneal injury or disease (e.g., alkali burn) models are used to study corneal wound healing, inflammation, and therapeutic agents for corneal diseases [95]. The model of C57BL/6 mice with induced dry eye (e.g., via lacrimal gland excision) mimics human dry eye conditions, allowing researchers to investigate inflammatory responses and treatment options [96]. Though mice have been explored to test nanomedicines for ocular diseases, their eyes are smaller than human eyes, which can complicate the translation of dosing and delivery methods. Additionally, some of their retinal and corneal structures differ from those in humans.

#### 3.4.2. Rat Model

Rat models are also widely used in ocular research, offering advantages like a larger eye size compared to mice, which facilitates certain surgical procedures, drug testing, and anatomical and behavioral studies. They are widely used due to their affordability, ease of handling, and established disease models that can mimic human eye conditions such as diabetic retinopathy, age-related macular degeneration, glaucoma, uveitis, corneal scar, and retinal ischemia reperfusion injury. STZ-induced diabetic rats develop hyperglycemia and retinal changes resembling human diabetic retinopathy, such as retinal vascular leakage, inflammation, and neurodegeneration [7]. A glaucoma model is induced by elevating IOP through episcleral vein cauterization or the injection of microbeads into the anterior chamber. These models lead to increased IOP and progressive damage to the optic nerve, mimicking glaucomatous changes seen in humans, such as retinal ganglion cell loss [97]. An AMD model could be generated via intravenous or intravitreal injection of NaIO3, which causes oxidative stress and degeneration of the retinal pigment epithelium (RPE) [98]. RCS rats have a mutation in the MerTK gene, leading to defective phagocytosis of photoreceptor outer segments by the RPE, causing progressive retinal degeneration similar to retinitis pigmentosa in humans [99]. An EAU-induced model mimics the immune-mediated inflammatory response in uveitis, with features like infiltration of inflammatory cells into the retina and vitreous body [100]. Retinal ischemia/reperfusion injury could be induced via transient elevation of IOP or occlusion of the retinal artery. This model mimics ischemic conditions that can occur in conditions like retinal vein occlusion, leading to retinal cell death and structural damage. It is useful for studying neuroprotection and therapies aimed at mitigating ischemic injury [101]. Corneal wound healing and neovascularization can be induced by mechanical or chemical injury (e.g., alkali burn) to the cornea. This model is used to study the mechanisms of corneal repair, neovascularization, and anti-inflammatory therapies, making it suitable for exploring treatments for corneal injuries and conditions like keratitis [102]. These models could be used for the evaluation of drug release profiles, toxicity, efficacy, and retinal targeting of nanomedicines through systemic or local routes such as intravitreal injections. However, rats’ eyes are smaller than human eyes, which can complicate the translation of dosing and delivery methods. Additionally, some of their retinal and corneal structures differ from those in humans.

#### 3.4.3. Rabbit Model

Rabbit models play a crucial role in ocular research due to their eye size, which is similar to that of humans, and their anatomical and physiological similarities, particularly in the anterior segment. Their large eyes make them especially useful for studying surgical interventions and drug delivery systems. Common rabbit models are used for various ocular diseases, such as glaucoma, corneal wound healing and neovascularization, dry eye disease, retinal detachment and degeneration, uveitis, and cataract formation. A glaucoma model is induced by laser photocoagulation of the trabecular meshwork or the injection of substances (e.g., α-chymotrypsin) into the anterior chamber. These methods lead to increased IOP, resulting in optic nerve damage and retinal ganglion cell loss, mimicking the pathology of human glaucoma [103,104]. The corneal wound healing and neovascularization model could be induced by mechanical, chemical (e.g., alkali burn), or surgical injuries to the cornea. This model is valuable for studying corneal epithelial wound healing, scarring, inflammation, and neovascularization, making it suitable for developing therapies for conditions like keratitis and corneal ulcers [105,106,107]. Dry eye disease induced by surgical removal of the lacrimal gland or the application of Benzalkonium chloride mimics the tear deficiency and ocular surface damage seen in dry eye conditions, enabling the study of tear film dynamics, inflammation, and therapeutic interventions [108,109]. Retinal detachment and degeneration models could be induced via subretinal injection of sodium hyaluronate or other agents. This model creates localized or widespread detachment of the retina, useful for studying the process of photoreceptor degeneration and evaluating potential therapies, such as neuroprotective agents [110]. Uveitis models induced by injecting endotoxins like lipopolysaccharides (LPSs) into the vitreous body or anterior chamber mimic the acute inflammatory response of uveitis, including cellular infiltration and inflammation of the anterior and posterior segments. They are useful for evaluating anti-inflammatory therapies [111]. Cataract induced by UV radiation or by injecting agents like selenite into young rabbits is used to study the pathophysiology of cataract formation and evaluate the effects of anti-oxidants, or surgical techniques for cataract removal can be explored to test new drugs.

Rabbits are commonly used in ocular pharmacokinetic studies due to their larger eye size, which is more comparable to that of humans. This makes them well suited for testing various formulations, such as eye drops and sustained-release implants [112,113].

#### 3.4.4. Non-Human Primate Model

Non-human primate (NHP) models are invaluable for studying ocular diseases due to their anatomical and physiological similarities to human eyes. These models bridge the gap between rodent studies and clinical trials, offering critical insights into disease mechanisms and therapeutic efficacy in a context closer to humans. Some key examples and their significance in ocular research are glaucoma, AMD, diabetic retinopathy, and uveitis. A glaucoma model is induced by laser photocoagulation of the trabecular meshwork or by applying microbeads to elevate IOP. These methods lead to increased IOP, causing optic nerve damage and retinal ganglion cell loss, mimicking human glaucoma pathology [114,115]. AMD models are developed using laser photocoagulation, which induces choroidal neovascularization (CNV) by causing breaks in the Bruch’s membrane due to high laser power. This approach creates conditions resembling wet AMD, enabling the study of anti-VEGF therapies and the evaluation of their impact on CNV and retinal structure preservation [116]. A DR model is induced by feeding species with Western diets or a galactose-rich diet, resulting in hyperglycemia and progressive retinopathy. These conditions mimic the vascular changes seen in human DR, allowing for the assessment of anti-angiogenic therapies and retinal laser interventions [117,118]. Uveitis models in primates are induced by introducing retinal S-antigen, leading to EAU, which mirrors the immune response seen in human uveitis. These models are valuable for testing immune-modulating therapies [119]. Non-human primates like rhesus monkeys are the gold standard for testing ocular drugs due to their close anatomical and physiological resemblance to the human eye, particularly the structure of the retina and macula. They are useful for testing advanced therapies, including gene delivery systems and intravitreal injections, for conditions like AMD and glaucoma. However, ethical considerations, high costs, and complex handling make NHP studies less common. They are typically reserved for late-stage preclinical research.

Accurate modeling is critical for bridging the gap between preclinical research and human clinical trials. Advanced in vitro cell culture models and in vivo animal models provide insights into how nanoparticles interact with ocular barriers like the cornea, blood–retina barrier, and vitreous humor. This knowledge helps predict the bioavailability of a drug in human eyes. For example, testing a sustained-release formulation in a rabbit model can help anticipate how long a drug remains active in the human eye.

## 4. Success Stories and Learnings

### 4.1. Case Studies of Translation of Nanomedicine from Preclinical to Clinical Trials

#### 4.1.1. Nanoparticle-Based Drug Delivery for Wet AMD (KSI-301)

KSI-301 is an anti-VEGF biologic conjugated to a biocompatible polymer, forming a nanoparticle that enhances ocular retention. Preclinical studies demonstrated that the nanoparticle formulation prolonged the duration of action in animal models, allowing for less frequent injections while maintaining efficacy in reducing retinal swelling and neovascularization. Based on these results, KSI-301 advanced to clinical trials, showing promise in extending the interval between treatments for patients with wet AMD. Early-phase clinical trials suggested that patients could maintain visual acuity with fewer injections compared to standard anti-VEGF therapies. The preclinical models were effective in demonstrating the benefits of extended drug release, a key advantage in treating chronic eye conditions like wet AMD. Aligning preclinical outcomes with clinical endpoints, such as prolonged therapeutic effects, played a critical role in successful translation [120].

#### 4.1.2. Brimonidine Drug Delivery System (Brimo DDS) for Geographic Atrophy in Dry AMD

A sustained-release formulation of brimonidine, delivered via a biodegradable intravitreal implant (Brimo DDS), was developed to provide neuroprotective effects in retinal diseases like geographic atrophy (GA). Preclinical models showed prolonged drug release and retinal neuroprotection in models of retinal degeneration. The results led to clinical trials assessing the ability of Brimo DDS to slow the progression of GA in patients with dry AMD. Although the clinical outcomes showed mixed results, the trials highlighted the potential of sustained-release nanomedicine platforms in ocular applications. The preclinical success of long-term drug release provided a strong rationale for clinical evaluation. However, the challenge of translating neuroprotective effects from animal models to humans underscores the need for disease models that better reflect human pathophysiology [121].

#### 4.1.3. Cyclodextrin-Based Nanoparticles for Ocular Inflammation (OCS-01)

OCS-01 is a dexamethasone-loaded nanoparticle using cyclodextrin technology designed to enhance drug penetration through ocular tissues, such as the cornea. Preclinical studies demonstrated improved drug solubility, sustained release, and effective anti-inflammatory effects in models of ocular inflammation. Positive preclinical results led to clinical trials for OCS-01 in treating DME and post-surgical inflammation. In Phase II clinical trials, OCS-01 demonstrated effective reductions in inflammation and macular thickness, supporting further development. The use of nanocarriers to enhance drug delivery to specific ocular tissues played a crucial role in successful translation. This case also highlights the importance of targeting formulations that address bioavailability challenges, which are often a hurdle in ophthalmic drug delivery [122].

### 4.2. Challenges and Key Takeaways from Successful and Failed Preclinical Trials

#### 4.2.1. Model Relevance

Successful preclinical studies often employ models that closely replicate human ocular anatomy and physiology, enabling more accurate prediction of therapeutic effects. Animal models that mimic conditions like AMD, diabetic retinopathy, or retinal inflammation are critical for evaluating both the efficacy and safety of nanomedicines [120,121,122].

#### 4.2.2. Clear Mechanistic Understanding

A deep understanding of how a nanomedicine interacts with ocular tissues and the mechanisms of drug release or targeting is crucial. Successful cases like KSI-301 leveraged a clear understanding of how polymer conjugation could extend drug action [120].

#### 4.2.3. Sustained Release and Dosing Flexibility

Preclinical studies that focus on optimizing sustained-release properties and determining the optimal dosing regimen can create a compelling case for clinical testing. This was evident in both KSI-301 and Brimo DDS, where the promise of fewer injections and prolonged therapeutic effects was a key factor [120,121].

#### 4.2.4. Mismatch Between Preclinical Models and Human Pathophysiology

Some failures, like those seen in neuroprotective strategies, often result from differences between animal models and human diseases. While animal models can suggest potential benefits, they may not fully replicate the progression and complexity of human conditions, leading to disappointing clinical results [123].

By learning from both successful and challenging cases, researchers can better design preclinical studies that are more predictive of clinical outcomes, ultimately advancing the development of nanomedicine therapies for ocular diseases.

## 5. Challenges in Clinical Translation

The development of new nanomedicine formulations and innovative applications for ocular diseases is rapidly advancing. While academic research and societal potential are striving for remarkable breakthroughs, there is growing scrutiny regarding industrial acceptance and the translation of these advancements into clinical practice. For successful translation of nanomedicines for treating ocular diseases, several key challenges need to be addressed: the pharmacokinetics (PK) and pharmacodynamics (PD) of nanomedicine in the eye, regulatory hurdles, manufacturing and scale-up, etc.

### 5.1. The Pharmacokinetics and Pharmacodynamics of Nanomedicines in the Eye

Ocular PK and PD focus on understanding how nanomedicines are absorbed, distributed, metabolized, and excreted within the eye, as well as their effects on ocular tissues [124]. Maintaining therapeutic drug levels in the eye presents several challenges due to its unique anatomical and physiological barriers. For instance, the corneal barrier, with its tightly packed epithelial cells, restricts the penetration of most drugs, especially larger, hydrophilic, and negatively charged molecules [125,126]. Similarly, the BRB formed by retinal endothelial cells and the RPE limits drug movement from systemic circulation to the retina. The blood–aqueous barrier adds another layer of restriction, making it difficult for drugs to reach the anterior segment of the eye [127]. These barriers, along with the rapid clearance of drugs through tear turnover and nasolacrimal drainage, often result in a short ocular half-life, requiring frequent dosing and leading to low patient compliance [125].

Achieving uniform drug distribution and prolonged retention in target tissues like the vitreous body or retina is also challenging due to the eye’s compartmentalized nature and its efficient clearance mechanisms. For example, drugs administered via intravitreal injection can be rapidly cleared through anterior or posterior pathways, limiting their duration of effect [128]. To overcome these challenges, increasing the drug dose may seem like an option, but it comes with risks such as inflammation, cytotoxicity, or elevated intraocular pressure. This risk is particularly significant in sensitive tissues like the retina, where damage can result in permanent vision loss, highlighting the need for precision in dosing strategies [129,130].

Nanoparticles offer promising solutions for overcoming these barriers and improving drug delivery to the eye. Their design, including size, surface charge, and hydrophilicity, influences their ability to penetrate ocular structures like the cornea, vitreous body, and retina. For instance, nanoparticles smaller than 200 nm with hydrophilic coatings are more likely to penetrate the conjunctival layers effectively, while those targeting the vitreous body must balance size and charge to navigate its gel-like structure. Polystyrene nanoparticles (PS NPs) as large as 510 nm rapidly penetrated the vitreous gel when coated with polyethylene glycol (PEG), whereas larger particles, such as those 1190 nm in diameter, were highly restricted regardless of surface chemistry due to steric obstruction. Additionally, PS NPs coated with primary amine groups (NH₂), which possess positively charged surfaces at the pH of bovine vitreous gel (pH 7.2), were immobilized within the vitreous gel [12,15,131,132]. Stability and controlled drug release are also critical; nanoparticles made from biodegradable materials must maintain their integrity in the eye to ensure sustained delivery. Encapsulation techniques can control release rates, but factors like particle interactions with ocular fluids and cellular uptake by retinal cells can affect delivery efficiency [133]. Additionally, minimizing immune responses through biocompatible coatings helps prolong the nanoparticles’ circulation time and reduce inflammation [134,135]. Understanding these factors is important for designing nanoparticle-based drug delivery systems that can maintain therapeutic levels and effectively treat ocular diseases.

### 5.2. Regulatory Pathways for Ocular Nanomedicine

Navigating the regulatory approval process for ocular nanomedicine is complex due to the unique challenges of the eye as a target organ and the novel nature of nanoscale drug delivery systems. The two primary regulatory agencies that guide the approval of these therapies are the U.S. Food and Drug Administration (FDA) and the European Medicines Agency (EMA) [136,137]. The regulatory approval of ocular nanomedicines by the FDA often comprises the following steps (i) identifying the target patient population, (ii) conducting preclinical studies, (iii) submitting an investigational new drug (IND) with clinical protocols for FDA review and clinical trial approval, and finally (iv) preparing and submitting a new drug application (NDA) with clinical results for FDA review, site inspection, and final approval [138,139]. For the European market, the process usually involves filing a clinical trial application (CTA) followed by a marketing authorization application (MAA) with the EMA [137]. Both pathways require a comprehensive dossier that includes data on preclinical studies, manufacturing processes, clinical trial results, and post-market surveillance plans.

Additionally, nanomedicine formulations often fall into a gray area between traditional drugs and combination products, which can further complicate the regulatory pathway. For instance, products that combine a drug with a delivery device, like a sustained-release implant for the eye, may need to meet additional regulatory criteria as combination products. This often involves coordinating with multiple FDA centers, such as the Center for Drug Evaluation and Research (CDER) and the Center for Devices and Radiological Health (CDRH), to determine the primary mode of action and appropriate regulatory pathway [136,140]. In Europe, the EMA’s Committee for Medicinal Products for Human Use (CHMP) plays a similar role, evaluating the balance between drug and device components in combined products [141].

### 5.3. Regulatory Challenges for Clinical Application of Ocular Nanomedicines

Ocular nanomedicines face several regulatory challenges compared to conventional drug molecules in clinical applications. The PK and PD characteristics of nanomedicines differ significantly from standard drug molecules due to the complex nature of nanomaterials, which vary in structure, shape, size, surface properties, and other physicochemical traits [140]. This complexity poses significant challenges for regulatory agencies in defining and classifying nanomedicines, particularly in determining standardized PK and PD profiles across different nanomedicine types. The lack of definitive protocols to assess nanotoxicity in various ocular layers is a major challenge. Standardized in vitro and in vivo protocols are essential, given the intricate structure and sensitivity of the human eye, to ensure accurate safety assessments of nanomedicines. Additionally, while enhanced permeability of nanomaterials can facilitate crossing of the blood–retina barrier, there is a risk of retinal accumulation, which may lead to toxicity in different retinal layers. Systemic accumulation can further impact normal ocular functions.

Currently, there is no unified regulatory framework for the clinical application of nanomedicines that is consistent across countries. Strict regulatory guidelines, interventions, and oversight at each stage of clinical application (as outlined in Figure 1) are essential to ensure the safety, efficacy, and quality of ocular nanomedicines before market approval.

### 5.4. Requirements for Demonstrating Safety, Efficacy, and Manufacturing Consistency

Both the FDA and EMA have stringent requirements for demonstrating the safety, efficacy, and manufacturing consistency of ocular nanomedicines. For safety, these agencies expect extensive preclinical studies, including acute and chronic toxicity assessments, detailed ocular histopathology, and PK data that show drug distribution and elimination profiles in the eye [136,139,140]. Toxicity studies must assess the impact of nanomaterials on critical ocular structures such as the retina, cornea, and optic nerve, with a focus on avoiding potential adverse reactions like inflammation or increased intraocular pressure.

Demonstrating efficacy is equally critical and requires robust clinical data that prove the therapeutic benefit of the nanomedicine in comparison to existing treatments. This includes clinical endpoints that are meaningful to patients, such as improvements in visual acuity or reductions in intraocular pressure for glaucoma treatments. Because the eye is relatively isolated from the systemic circulation, regulatory bodies often require that efficacy studies in humans align closely with PK and PD data obtained from preclinical models. This alignment helps ensure that the drug achieves therapeutic levels in the targeted ocular compartments without significant systemic exposure.

Manufacturing consistency is a particularly challenging aspect for nanomedicines because subtle variations in the production process can significantly impact the physicochemical properties of the nanoparticles, such as size, shape, drug-loading capacity, and release profile [15]. The FDA and EMA require that manufacturing processes be developed and validated to ensure batch-to-batch consistency. This includes establishing strict Good Manufacturing Practice (GMP) guidelines for the scale-up of nanoparticle production, stability testing, and sterility assurance for formulations intended for ocular use [136]. Nanomedicines often need specialized analytical techniques to verify the quality of the final product, such as dynamic light scattering for particle size analysis or high-performance liquid chromatography (HPLC) for drug content determination [7,142].

Overall, the regulatory hurdles for ocular nanomedicines are rigorous due to the need to balance innovation with patient safety. Developers must navigate these requirements by demonstrating robust safety and efficacy data while ensuring that manufacturing processes produce high-quality, reproducible products. Early engagement with regulatory bodies is crucial to clarify requirements and streamline the approval process for these advanced therapies.

### 5.5. Manufacturing and Scale-Up

Scaling up the production of nanoparticles from laboratory research to commercial manufacturing presents several challenges. Ensuring stability and reproducibility are crucial, as maintaining consistent nanoparticle characteristics—such as size, shape, surface charge, and drug encapsulation efficiency—can be difficult during large-scale production. Small variations in process parameters, like temperature and mixing speed, can significantly affect nanoparticle properties, impacting their safety and efficacy. Quality control is also essential, requiring robust analytical methods to monitor key attributes and ensure batch-to-batch consistency. Additionally, high production costs due to specialized equipment, raw materials, and quality control processes pose economic challenges [143,144]. Addressing these issues through process optimization and advanced manufacturing techniques is vital for the successful clinical translation of nanoparticle-based therapies.

### 5.6. Clinical Trial Design

Designing clinical trials for nanomedicines requires careful consideration of several key factors to ensure safety, efficacy, and regulatory approval [42,145,146]. Clinical trials for ocular diseases face several potential challenges, including issues with patient selection, randomization, and assessment of the placebo effect. Patient selection is crucial, as it involves identifying appropriate populations based on disease severity, prior treatments, and specific conditions that could influence the interaction of nanoparticles with biological systems. For instance, patients with ocular hypertension or open-angle glaucoma (OAG), including those with secondary forms such as pseudoexfoliation (PXG) or pigmentary dispersion glaucoma (PDG), may respond differently to treatments, raising questions about how their inclusion could affect therapeutic outcomes [146,147]. Patients with varying stages of a disease or specific genetic markers may respond differently to nanoparticle-based therapies, making targeted selection essential to assess therapeutic outcomes effectively [147,148]. Endpoints must be clearly defined, with a focus on both efficacy (e.g., improvement in visual acuity for ocular diseases) and safety (e.g., monitoring inflammation, adverse reactions, or changes in intraocular pressure). Depending on the route of administration, such as topical, intravitreal, or subconjunctival, endpoints may vary to capture relevant therapeutic effects and potential side effects [149,150].

Study design is tailored to the nature of the formulation and the disease being treated. Topical formulations, often used for ocular applications, may focus on endpoints like local bioavailability and reduced systemic absorption to minimize side effects. The trial design may include crossover studies where patients serve as their own controls, allowing for comparison of the topical treatment’s efficacy. For example, in clinical trials of dry eye diseases, the endpoints that could be considered are the ocular surface disease index score, tear break-up time, tear production, and the presence of corneal punctate lesions [151,152,153]. Intravitreal formulations, used for conditions like AMD or DR, require more rigorous monitoring of localized effects, including potential risks such as retinal toxicity or increased IOP. These trials often involve careful dose titration studies and extended follow-up periods to assess long-term safety and efficacy [154,155]. Each formulation type requires unique considerations in trial design to ensure that the nanomedicine achieves the intended therapeutic effect while minimizing risks, ultimately supporting a successful path to clinical use.

## 6. Safety and Toxicity Considerations

### 6.1. Biocompatibility of Nanomaterials in Ocular Applications

Biocompatibility is a critical factor when developing nanomaterials for ocular applications, as the eye contains delicate tissues that are sensitive to foreign materials. This includes evaluating the interaction of nanoparticles with different ocular structures like the cornea, conjunctiva, vitreous humor, and retina. The safety profile of nanoparticles is assessed through studies that measure oxidative stress, cellular viability, tissue integrity, and the absence of inflammatory responses, in both in vitro (e.g., using corneal or retinal cell lines) and in vivo models (e.g., using rodent or rabbit eyes), retinal function, and visual acuity in vivo [4,7,142]. For instance, biodegradable biocompatible polymers are often preferred for ocular applications due to their well-documented safety profiles and ability to degrade into non-toxic byproducts [15,156].

To minimize toxicity and inflammatory responses, surface modification of nanoparticles is often employed. For example, coating nanoparticles with polyethylene glycol (PEG) (a process known as PEGylation) can reduce protein adsorption and minimize immune recognition, leading to a better biocompatibility profile [134,157]. Additionally, using anti-inflammatory coatings, such as dexamethasone-loaded nanoparticles, can provide therapeutic effects while also minimizing the potential inflammation triggered by the nanoparticles themselves [158,159]. Adjusting the surface charge of nanoparticles is another strategy, as highly positively charged particles tend to cause more irritation or toxicity compared to neutral or slightly negative ones [160,161]. Balancing these design parameters helps in reducing unwanted immune reactions and ensuring that the nanoparticles can be safely used for sustained drug delivery within the eye.

Different types of nanoparticles, including liposomes, polymeric nanoparticles, and metallic nanoparticles, can elicit varying degrees of immune responses when administered into the eye. The eye’s immune privilege—its ability to limit inflammatory responses to maintain visual clarity—can be disrupted by the introduction of nanoparticles, leading to conditions like uveitis or increased intraocular pressure [162]. For example, metallic nanoparticles (e.g., gold or silver) may induce higher levels of oxidative stress and inflammation compared to biodegradable polymeric carriers [163]. The immune response is influenced by the physicochemical properties of the nanoparticles, such as size, shape, surface charge, and material composition [164]. Understanding these interactions allows for the design of nanoparticles that minimize immunogenicity while maintaining therapeutic efficacy.

### 6.2. Understanding the Mechanisms of Nanoparticle Clearance from Ocular Tissues

Clearance mechanisms for nanoparticles in the eye vary depending on the route of administration and the targeted ocular compartment [67,150]. For topical applications, nanoparticles can be cleared through tear turnover, nasolacrimal drainage, or absorption into the systemic circulation [165]. For intravitreal injections, nanoparticles are primarily cleared through the trabecular meshwork into the systemic circulation or through phagocytosis by retinal cells and resident immune cells like microglia [150,166]. The size and surface characteristics of nanoparticles significantly influence their retention time; for example, negatively charged nanoparticles (NPs) smaller than 100 nm exhibit high diffusion rates within the vitreous body. However, they are quickly cleared through the anterior route via aqueous humor drainage and the posterior route, especially for those smaller than 2 nm [166]. Designing nanoparticles that balance sustained drug release with appropriate clearance rates is crucial to maintaining therapeutic levels while minimizing potential long-term accumulation and toxicity in ocular tissues.

### 6.3. Case Studies of Safety Profiles in Clinical Trials

Review of Safety Data from Clinical Trials of Leading Nanomedicine Formulations: Clinical trials of nanomedicine formulations targeting ocular diseases provide crucial insights into the safety and biocompatibility of these technologies. Phase I/II clinical trials are majorly evaluating the safety and biocompatibility drugs or formulations [146,147,167]. The primary endpoints include visual comfort, assessed using the Ocular Comfort Index (OCI), and safety, which was evaluated through laboratory tests, vital signs (VSs), visual acuity (VA), IOP, lissamine green and fluorescein staining, conjunctival hyperemia, chemosis, and the incidence of adverse events (AEs) [167]. However, for intravitreal injection, the safety data generally focus on adverse events such as inflammation, increased intraocular pressure, or retinal detachment, as well as monitoring for systemic effects [154,155]. In these trials, researchers found that using a nanoparticle-based formulation can prolong drug release and reduce the frequency of injections, which is particularly beneficial for patients with chronic conditions like DR and AMD. However, monitoring for inflammation or immune responses to the nanoparticles is critical, as even mild reactions could compromise vision.

Another example is dexamethasone intravitreal implants, which utilize biodegradable polymer-based technology to treat macular edema and non-infectious uveitis. Clinical trials demonstrated that the sustained release provided by these implants significantly reduced the need for repeat injections while maintaining a stable safety profile, making it a preferred option for patients. Common side effects reported included increased intraocular pressure and cataract formation, which were manageable with appropriate clinical interventions. The data gathered in these trials highlight the importance of balancing therapeutic benefits with potential side effects, ensuring that patients receive effective long-term treatment without compromising ocular health [168,169,170].

Patient feedback from clinical trials of ocular nanomedicine formulations often emphasizes the benefits of reduced treatment burden. For example, participants in trials for sustained-release formulations have noted a preference for treatments that minimize the frequency of invasive procedures like intravitreal injections, as these can be both physically uncomfortable and logistically challenging. This feedback is especially relevant for conditions like diabetic macular edema or AMD, where patients often require frequent follow-up visits and repeat dosing over many years. Patient-reported outcomes, such as visual acuity improvement and reductions in symptoms like pain or discomfort during administration, help to assess the overall acceptance of new therapies [171,172,173,174].

However, patient perspectives also highlight concerns about potential side effects, such as transient blurriness or irritation after nanoparticle-based topical applications, as well as the risk of serious complications like retinal detachment following intravitreal injections. These concerns underscore the need for transparent communication during trials about possible risks and long-term safety. The incorporation of patient feedback into the design of future studies can guide the optimization of delivery methods and formulations, ensuring that new therapies meet both clinical efficacy and patient satisfaction criteria, and can illustrate how safety data from clinical trials are integral to assessing the feasibility of new nanomedicine formulations for ocular diseases. Patient feedback not only informs the regulatory approval process but also plays a key role in refining therapeutic strategies for better acceptance in real-world clinical settings [31,174,175].

## 7. Strategies for Successful Translation

### 7.1. Designing Clinically Relevant Formulations

Designing clinically relevant formulations for ocular diseases requires a strategic approach that bridges the gap between early-stage research and clinical implementation. Early formulation design must account for the unique anatomy and physiology of the eye, including barriers like the tear fluid barrier, tight junctions of the corneal and conjunctival epithelium, blood–ocular barrier, and blood–retina barrier. This helps in selecting appropriate drug delivery systems that can effectively deliver therapeutics to specific ocular tissues [12,15]. Among these systems, liposomes and other nanocarriers have shown significant promise. For example, liposome-based delivery systems provide a novel alternative to intravitreal injections by encapsulating drugs like triamcinolone acetonide (TA), the most frequently used intraocular synthetic corticosteroid. These nanoparticle-based systems offer potential for treating posterior segment diseases with improved patient compliance and reduced invasiveness [176]. Nanocarriers, including liposomes, polymeric nanoparticles, and micelles, have demonstrated the ability to enhance the intraocular half-life and bioavailability of various therapies [177]. Liposomes, in particular, are the most utilized nanocarriers for ocular drug delivery due to their biocompatibility, ability to encapsulate both hydrophilic and hydrophobic drugs, and potential for controlled release [178].

The next step is to ensure that the drug is stable in the chosen formulation and remains active over time, which is crucial. For ocular delivery, ocular retention, and penetration, compatibility with ocular tissues is critical for achieving effective therapeutic concentrations [7,179]. Further, scalability and manufacturing feasibility are very important. Formulations should be designed with a future perspective of scale-up and GMP (Good Manufacturing Practice) production, because subtle variations in the manufacturing process of nanomedicines can significantly impact their physicochemical properties, including size, shape, composition, crystallinity, drug-loading capacity, drug release profile, and surface functionality and chemistry. Therefore, methods like nanoparticle synthesis, emulsions, or liposome preparation should be adaptable to large-scale manufacturing without significant changes in drug release profiles or stability [180]. Addressing regulatory requirements early on, including selecting excipients and delivery materials that are approved for ophthalmic use, can streamline the path to clinical trials. Understanding guidelines from regulatory bodies like the FDA or EMA ensures that the formulation design aligns with safety standards and accelerates the translation process.

Multidisciplinary collaboration between scientists, clinicians, and engineers is crucial for the successful development of ocular nanomedicine. Pharmacologists bring insights into drug mechanisms and targeting, ophthalmologists provide an understanding of disease pathology and clinical needs, and biomedical engineers design innovative delivery platforms that address these needs. For example, the development of nanocarrier systems like liposomes or polymeric nanoparticles for retinal diseases benefits from the combined expertise of materials scientists (for carrier design), ophthalmologists (for clinical relevance), and pharmacokinetic experts (for drug release dynamics). Furthermore, early collaboration between preclinical researchers and clinical trial experts ensures that animal models and testing protocols closely mirror human disease conditions, making the transition from bench to bedside smoother as the formulation’s efficacy and safety are evaluated in models that better predict human responses. Collaborating with patient advocacy groups or directly with engaging patients during the design phase can also inform practical considerations like ease of use, dosing frequency, and preferences, ultimately improving adherence and treatment outcomes in clinical settings. Challenges such as maintaining therapeutic drug levels or achieving targeted delivery to specific ocular tissues (e.g., retina or choroid) often require innovation at the intersection of materials science, pharmacology, and clinical practice. Addressing these through multidisciplinary teams fosters solutions that are both scientifically sound and clinically practical. An early-stage focus on clinical applicability, paired with collaboration across disciplines, is important for designing formulations that overcome the challenges of ocular drug delivery and successfully navigate the complexities of clinical translation.

### 7.2. Overcoming Delivery Barriers

Overcoming delivery barriers in the eye is essential for effective treatments, given the complex anatomy and protective mechanisms that limit drug access to ocular tissues. Modifying the surface of nanoparticles with ligands (e.g., proteins, peptides, carbohydrates, antibodies) that bind to specific receptors on corneal or retinal cells can enhance targeted delivery, such as using hyaluronic acid-coated nanoparticles to target CD44 receptors on corneal epithelial cells, improving retention time on the eye surface [4,181], and controlling the physicochemical properties of the nanoparticles for their spatiotemporal biodistribution in the eye [12,15]. Nanoparticles designed within a specific size range (typically < 200 nm) can better penetrate through ocular barriers like the tight junctions of the corneal epithelium or the blood–retina barrier, while adjusting the shape (e.g., rod-shaped, triangle-shaped, spherical) can influence their interaction with ocular surfaces and their ability to diffuse through dense tissues like the vitreous humor [12,15,133,182]. Utilizing biodegradable polymers (e.g., PCL, PLA, PLGA,) allows for the development of nanoparticles that provide controlled and sustained release of drugs over weeks or even months, which is particularly advantageous for treating chronic conditions like diabetic retinopathy, glaucoma, or AMD, where frequent dosing is a challenge [11,183,184]. To make use of stimuli such as temperature, pH, enzymes, and light, stimulus-responsive drug delivery systems could be engineered to target and release drugs in various ocular compartments [4,13,185]. Additionally, lipid-based nanoparticles like nanostructured lipid carriers (NLCs) and solid lipid nanoparticles (SLNs) can merge with lipid-rich structures in the eye, such as the corneal epithelium, facilitating enhanced absorption and retention, while their ability to encapsulate both hydrophilic and hydrophobic drugs, coupled with their biocompatibility and protection of sensitive drugs from degradation, makes them versatile options for ophthalmic formulations [186].

### 7.3. Engagement with Regulatory Agencies

Engaging with regulatory agencies early in the development of ocular drug delivery systems is essential for navigating the complex approval process and ensuring that formulations meet safety and efficacy standards. Here are the key best practices and strategies to align preclinical data with clinical requirements.

#### 7.3.1. Best Practices for Early Communication with Regulatory Bodies

Initiating a Pre-Investigational New Drug (Pre-IND) meeting with agencies like the FDA or EMA can provide early guidance on study design, regulatory requirements, and expectations for preclinical data. This helps ensure that the drug development program is on the right path from the beginning. In these meetings, developers can discuss proposed formulations, novel delivery technologies, potential challenges, and planned preclinical studies. Early feedback can significantly streamline the process by identifying and addressing potential regulatory concerns at an early stage. Regulatory agencies often have specific guidance documents for ophthalmic products, addressing unique aspects like ocular toxicity, delivery routes (topical, intravitreal, etc.), and safety considerations for ocular tissues [187,188].

For instance, the FDA’s guidance documents like “Development of New Topical Ophthalmic Drug Products” provide detailed recommendations for conducting ocular toxicity studies and assessing systemic exposure. Aligning with such guidelines helps in designing studies that meet regulatory expectations and minimize the risk of additional data requests. Ongoing communication beyond the initial meetings can prevent delays and misunderstandings. As development progresses, sharing updates with the regulatory body—especially when there are significant changes in formulation design, delivery methods, or preclinical data—can keep the process aligned. Utilizing written communications, such as the FDA’s Controlled Correspondence mechanism, for specific queries regarding formulation components or study protocols can help clarify doubts and set clear expectations. Programs like the FDA’s Orphan Drug Designation or the EMA’s PRIME (PRIority MEdicines) can be beneficial for drugs targeting rare or serious conditions, including those for ocular diseases with limited treatment options. These programs provide accelerated guidance and review pathways, helping to expedite the development process [189].

#### 7.3.2. Strategies for Aligning Preclinical Data with Clinical Requirements

Using animal models that closely mimic human ocular diseases is crucial for generating data that can predict human responses. Selecting the right species or model for testing intraocular formulations is important, as certain species may better represent human eye anatomy or disease progression. Ensuring that preclinical models simulate the intended clinical route of administration provides more accurate data on drug distribution, pharmacokinetics, and potential side effects—critical aspects that regulatory bodies will scrutinize. Conducting Good Laboratory Practice (GLP)-compliant safety studies is mandatory before entering clinical trials, including acute and chronic toxicity studies in two species (typically a rodent and a non-rodent) to assess both ocular and systemic safety. Regulatory agencies focus on potential damage to critical ocular structures like the cornea, retina, optic nerve, and lens, so including detailed ophthalmic exams, electroretinograms (ERGs), and histopathology data is essential for building a comprehensive safety profile. Understanding the long-term behavior of nanoparticles in ocular tissues, including their degradation and clearance, is also crucial to prevent toxic accumulation.

Collecting robust PK and PD data in preclinical studies helps demonstrate how the drug behaves in ocular tissues and supports dosing strategies for clinical trials. Regulatory agencies require detailed data on drug concentration over time in target ocular compartments. It is critical to design studies that show that the therapeutic levels achieved in preclinical models are likely to be replicable in humans, aligning with the target product profile and proposed clinical endpoints. Bioavailability studies comparing novel formulations with existing therapies can provide valuable insights, highlighting the advantage of a new delivery system in achieving desired drug levels. A well-prepared IND or CTA dossier includes preclinical study results and detailed information on the formulation, manufacturing process, stability data, and analytical methods used. Careful documentation of data quality and study protocols and a thorough risk assessment of the formulation can reduce queries from regulatory bodies and expedite the approval process. For novel drug delivery systems, emphasizing their ability to address unmet needs in ocular drug delivery, supported by robust preclinical data, can strengthen the regulatory submission and highlight its clinical benefit. By following these best practices for early engagement and aligning preclinical data with clinical goals, developers can smooth the path to regulatory approval, increasing the likelihood of successful clinical translation for innovative ocular drug delivery systems.

## 8. Future Directions and Opportunities

### 8.1. Emerging Trends in Ocular Nanomedicine

Emerging trends in ocular nanomedicine are focusing on advanced approaches like gene therapy and RNA-based therapies, which are delivered using nanoparticles to achieve precise, localized effects in treating eye diseases [190,191,192]. These therapies utilize nanoparticles to safely deliver RNA directly to target cells in the eye, potentially offering more effective and sustained treatments for conditions like AMD or corneal fibrosis [193,194]. Additionally, the development of stimuli-responsive and targeted delivery systems is gaining traction. These systems can respond to specific triggers, such as changes in pH, temperature, or light, to release drugs in a controlled manner at the site of action [4]. This targeted approach not only improves the efficacy of therapeutic agents by concentrating their effects in the desired ocular tissues but also minimizes systemic exposure and potential side effects, making these technologies promising candidates for future treatments in ocular diseases.

### 8.2. Integrating Nanomedicine with Digital Health

Integrating nanomedicine with digital health is opening new avenues for precision medicine, especially in optimizing therapeutic approaches and monitoring patient outcomes. The use of artificial intelligence (AI) and machine learning (ML) in the design of nanoparticle formulations allows for the analysis of vast datasets to predict the most effective particle size, surface properties, and drug release profiles for specific conditions [195,196,197,198]. These technologies can identify optimal formulations more quickly than traditional trial-and-error methods, potentially reducing the time and cost of drug development. Furthermore, digital monitoring tools, such as wearable sensors and mobile health applications, are increasingly being used to track patient responses and assess the efficacy of nanomedicine treatments in real time during clinical trials. These tools can collect continuous data on physiological parameters, patient-reported outcomes, and adherence to treatment protocols, providing a comprehensive picture of how therapies are working in real-world settings [199]. This integration of AI-driven formulation design and digital monitoring enhances the ability to personalize treatments, adjust therapies promptly, and improve overall patient outcomes in nanomedicine.

### 8.3. Perspectives on Personalized Nanomedicine

Personalized nanomedicine offers a promising approach for tailoring treatments based on individual genetic and molecular profiles, providing opportunities for more precise and effective therapies. By integrating advanced diagnostics like genomic sequencing and proteomics, it is possible to identify specific biomarkers associated with a patient’s disease state and use this information to customize nanomedicine formulations [200,201,202]. For example, in ocular diseases, nanoparticles can be engineered to deliver drugs directly to retinal cells expressing certain genetic markers, thereby increasing drug efficacy and minimizing side effects. Similarly, in conditions like glaucoma or AMD, nanocarriers can be designed to respond to the unique physiological environment of each patient’s eye. This approach enables more targeted delivery, controlled drug release, and reduced systemic exposure, ultimately improving treatment outcomes. The potential for personalized nanomedicine extends beyond treatment to include monitoring disease progression and adjusting therapies in real time through digital health technologies, ensuring that patients receive therapies that are not only effective but also tailored to their specific biological make-up [200].

## 9. Conclusions

The journey to the clinical translation of ocular nanomedicine has highlighted several key insights, including the importance of precise drug delivery, sustained-release formulations, preclinical testing of drugs in appropriate models, and patient-centered design in improving therapeutic outcomes for eye diseases. Despite significant progress, challenges remain, such as the complexities of navigating regulatory pathways, ensuring long-term safety, and optimizing the retention of nanoparticles in ocular tissues. Addressing these obstacles is crucial for the broader adoption of nanomedicine in clinical practice. There is a tremendous opportunity to advance the field through collaborative efforts between researchers, clinicians, and industry, which can accelerate innovation and overcome barriers to translating promising preclinical findings into effective clinical therapies in eye care. A call to action for interdisciplinary collaboration aims to drive the next generation of breakthroughs, ultimately improving patient care and expanding the impact of nanomedicine in ophthalmology.

## Figures and Tables

**Table 1:** FDA-approved nanomedicine for ocular diseases.

Nanomedicine Approved by FDA	Diseases	Year of FDA Approval	Types of Formulation and Mechanism of Action
Visudyne^®^	AMD	2000	It is a liposomal formulation of verteporfin. It is used in photodynamic therapy (PDT) to target abnormal blood vessels in the eye [25].
Restasis^®^	Chronic dry eye	2003	It is an oil-in-water nanoemulsion of cyclosporine A. The nanoemulsion improves the bioavailability of cyclosporine on the ocular surface [23].
Cequa^®^	Dry eye disease	2018	It is a nanomicellar formulation of cyclosporine that enhances its penetration through the aqueous layer of the tear film [26].
AzaSite^®^	Bacterial conjunctivitis	2007	It uses a Polycarbophil mucoadhesive delivery system which stabilizes and sustains the release of azithromycin to the ocular surface by forming a stable mucoadhesive matrix that stays in contact with the conjunctiva [27].
Durezol^®^	Post-operative inflammation and pain	2008	It is an oil-in-water lipid nanoemulsion of Difluprednate, a corticosteroid, which improves intraocular penetration [28].
Systane^®^	Dry eye disease	2018	It is an anionic nanoemulsion leading to pH-dependent gelling (agent-hydroxypropyl -guar), resulting in prolonged ocular contact of demulcents [29].
EYSUVIS^®^	Dry eye disease	2020	It is a nanosuspension formulation of loteprednol etabonate which uses mucus-penetrating particles, resulting in enhanced penetration of loteprednol etabonate into target tissue on the ocular surface [30].
Cationorm^®^	Dry eye disease	2008	It is a positively charged (due to cetalkonium chloride) nanoemulsion which prolongs the residence time of mineral oils, surfactants, and glycerin on the slightly negative ocular cellular surface, resulting in stabilization of the tear film and hence a long-lasting soothing effect [31].
Trivaris™	Uveitis	2008	It is a sodium hyaluronate microparticle system releasing triamcinolone acetonide to reduce inflammation [32].
Xelpros^®^	Open-angle glaucoma or high intraocular pressure	2018	It uses an oil-in-water microemulsion (polymer/castor oil micelle) of latanoprost, a prostaglandin analog, resulting in easier penetration of latanoprost along with supplementation of the tear film [33]. Latanoprost increases fluid outflow from the eye, leading to the lowering of ocular pressure.
Verkazia^®^	Vernal keratoconjunctivitis	2021	It is an oil-in-water cationic emulsion of cyclosporine, improving its bioavailability and hence leading to the inhibition of T-cell activation and a reduction in inflammation at the ocular surface [34].
Besivance^®^	Ocular bacterial infection and bacterial conjunctivitis	2009	It uses Durasite^®^ technology (similar to AzaSite^®^) for the delivery of besifloxacin, a fluoroquinolone, leading to improved bioavailability up to 12 h [35].
Tobradex ST^®^	Ocular inflammation and bacterial infection	2009	Nanosuspension of the antibiotic tobramycin and the corticosteroid dexamethasone which reduces bacterial growth and inflammation [36,37].
Triesence^®^	Ocular inflammatory conditions non-responsive to topical corticosteroids, uveitis, sympathetic ophthalmia, and temporal arteritis	2007	A microneedle-based system delivering triamcinolone acetonide resulting in increases in vitreal residence [38].

**Table 2:** Clinical trials of nanomedicines for ocular diseases.

Nanomedicine	Phase of Clinical Trial	Ocular Diseases	Application and Clinical Trial Identifier Number
Lytenava™	II (completed)	DME and neovascular AMD	It involves intravitreal injection of a small molecule, UBX1325, inhibiting Bcl-xL and targeting cellular senescence, implicated in retinal diseases, aiming to improve retinal function and reduce vascular leakage [39] (NCT04857996).
THR-149	II (completed)	DME	It is a nanoparticle-based formulation for intravitreal injection. It works by blocking plasma kallikrein, which is involved in inflammation and vascular permeability in DME, thus aiming for improved control of macular edema [40] (NCT04527107).
RGX-314	III (recruiting)	Neovascular AMD	It is an AAV8-based gene therapy delivered via subretinal injection. Designed to provide long-term anti-VEGF expression (ranibizumab-like protein) directly in the retina, it reduces the need for frequent anti-VEGF injections [41] (NCT04704921, NCT05407636).
KSI-301	III (completed)	DR, retinal vein occlusion (RVO), DME, and wet AMD	It is a conjugate of an anti-VEGF antibody with a biopolymer for sustained ocular delivery. It provides prolonged VEGF inhibition, allowing for less frequent dosing [42] (NCT04592419).
Ixo-vec (formerly ADVM-022),	I (completed)	Wet AMD	It is an AAV-based gene therapy approach delivered via intravitreal injection, resulting in the long-term, stable expression of therapeutic anti-VEGF, aflibercept [43] (NCT03748784).
4D-150	II (recruiting)	Wet AMD	It is a gene therapy-based approach and involves an intravitreal injection of an AAV vector encoding aflibercept and an miRNA sequence targeting VEGF-C, aimed toward patients who require frequent anti-VEGF therapy [44] (NCT05197270).
OCS-01	III (recruiting)	DME	It is a topical nanoparticle-based eye drop formulation for enhanced corneal penetration aiming to deliver therapeutic levels of dexamethasone to the retina non-invasively [45] (NCT05066997).
Xipere^®^	III (completed); FDA approval received	Macular edema associated with uveitis	Micronized triamcinolone is delivered via suprachoroidal injection using a specialized applicator targeting the suprachoroidal space to reduce inflammation and edema directly at the site of pathology [46] (NCT02595398).

## Data Availability

Not applicable.
